# Nutrition and Hypertension Researches in 2023: focus on salt intake and blood pressure

**DOI:** 10.1038/s41440-024-02089-5

**Published:** 2025-01-27

**Authors:** Chisa Matsumoto

**Affiliations:** 1 Center for Health Surveillance & Preventive Medicine, Tokyo Medical University Hospital, Tokyo, Japan; 2 Department of Cardiology, Tokyo Medical University, Tokyo, Japan

**Keywords:** Nutrition, Salt intake, Salt substitution, Magnesium, Vitamin E, RES

## Abstract

Hypertension is a major global health issue that contributes significantly to cardiovascular morbidity and mortality. The management and prevention of hypertension often involve nutritional and dietary modifications, which are considered effective non-pharmacological strategies. In 2023, the Hypertension Research published several papers highlighting nutrition and hypertension. In addition, multiple studies published in leading journals explored the relationship between salt intake and blood pressure (BP) in 2023. In this mini-review, we summarize the key findings of nutritional studies published in the Hypertension Research in 2023. This mini-review also highlights significant findings from the latest research on salt intake and its impact on BP. The new findings from nutritional studies will provide deeper insights on planning dietary strategies for the management of hypertension.

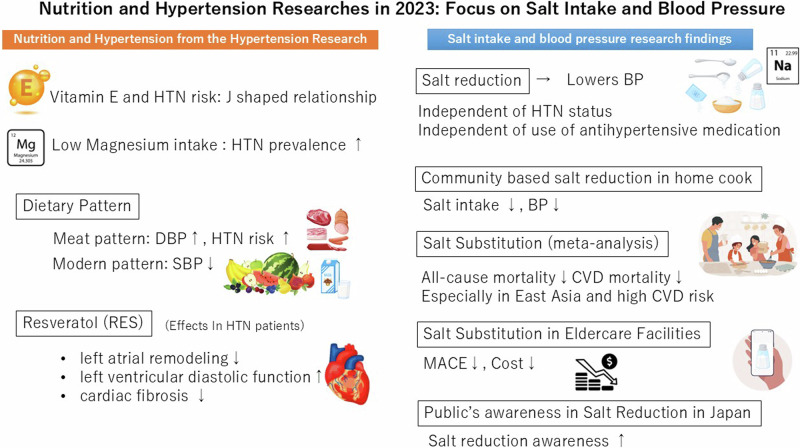

## Update on Nutrition and Hypertension: from the Hypertension Research in 2023

Recent studies have suggested a potential correlation between various micronutrients and the risk of hypertension. Among these, the relationship between magnesium intake and risk of hypertension was reported in 2023 [1]. Although the literature on the association between magnesium consumption and lifestyle-related diseases is relatively sparse, Han et al. examined the association between magnesium intake, assessed through 24-hour dietary recall, and the prevalence of hypertension, diabetes, and dyslipidemia using data from NHANES 2007–2018, a nationally representative cross-sectional survey of health in the United States corresponding to 34,700 adult participants [1]. Their analysis revealed an inverse relationship between magnesium intake and prevalence of all three conditions. Notably, individuals in the highest quintile of magnesium intake exhibited a 34% reduction in the prevalence of hypertension compared with those in the lowest quintile (odds ratio [OR] 0.66, 95% confidence interval [CI] 0.51–0.87, *P* trend < 0.001) [1]. As, meta-analyses of previous randomized controlled trials (RCTs) have also suggested a blood pressure (BP)-lowering effect of magnesium supplements, particularly in high-quality RCTs with an increase in serum magnesium levels followed by magnesium supplementation [2], this evidence suggest causal relationship between magnesium intake and the reduction of BP. The mechanism of the BP-lowering effects of magnesium is uncertain. However, extracellular Mg^2+^ has been reported to inhibit capacitative Ca^2+^ entry into vascular smooth muscle cells, which may determine vascular contractility and modulate BP [3]. Also, potential antihypertensive effect of magnesium by the modulation of vascular smooth muscle cell function, reduction in peripheral vascular resistance, and antioxidant effects have been reported [4–6].

As one of micronutrients, the relationship between vitamin E intake and hypertension was also explored [7]. Vitamin E is predominantly found in plant-based foods such as nuts, vegetable oils, and certain fruits and vegetables, as a lipid-soluble antioxidant essential for human health. It has been postulated that vitamin E may support or enhance vascular endothelial function through various mechanisms [8, 9]. Supporting this hypothesis, a previous meta-analysis of 18 RCTs involving 839 participants indicated that vitamin E supplementation was associated with a reduction in systolic blood pressure (SBP) [10]. However, it is important to note that these RCTs primarily investigated the effects of short-term and high-dose vitamin E supplementation (80 to 1206 mg/day and intervention durations ranging from 3 to 48 weeks), with substantial heterogeneity across studies (I^2^ = 94.0%, *P* < 0.001) [10]. In a more extensive study, Zhang et al. examined the long-term association of vitamin E intake and hypertension using data from the China Health and Nutrition Survey, which included 12,177 participants (mean age, 41.2 ± 14.2 years) [7]. Vitamin E intake was assessed using a 24-hour dietary recall method and a median energy-adjusted dietary vitamin E intake was 27.5 mg/day interquartile range (IQR) : 20.3–37.5 mg/day) [7]. During a median follow-up of 6.1 years, 4269 individuals developed hypertension. The analysis revealed a J-shaped relationship between vitamin E intake and the risk of developing hypertension, even after adjusting for other major nutrients or major food groups, including vegetables, fruits, legumes, grains, nuts, and vegetable oils. Participants in the lowest quintile of dietary vitamin E intake (< 18.75 mg/day) exhibited a significantly higher risk of developing hypertension than those in the second to fourth quintiles (18.75 to < 40.53 mg/day), with an adjusted hazard ratio (HR) (95% CI) of 1.40 (0.51–0.87). On the contrary, participants in the highest quintile of dietary vitamin E intake (≧ 40.35 mg/day) revealed a significantly higher risk of developing hypertension compared to those in the second to fourth quintiles (adjusted HR 1.18, 95% CI 1.09–1.29) [7]. Although the precise mechanisms are not yet fully understood, these findings are biologically plausible. Vitamin E, a potent peroxyl radical scavenger, mitigates oxidative stress, improves endothelial function, and increases the production of vasodilatory prostanoids prostaglandin I2 and E2 [11, 12]. However, high doses of vitamin E may have pro-oxidant effects, impair endothelium-dependent arterial relaxation, and disrupt the balance of antioxidant systems, potentially increasing susceptibility to oxidative damage [13–15]. Therefore, maintaining optimal dietary vitamin E intake levels could lead to the prevention of hypertension.

Polyphenols, particularly resveratrol (RES), a compound abundant in berries, grapes, rhubarb, and peanuts, have been suggested to possess cardiovascular protective effects. Although animal studies have demonstrated the cardiovascular benefits of RES [16–18], evidence in humans has been limited. Zueng et al. conducted a study on 80 hypertensive patients to investigate whether supplementation with 400 mg RES could offer additional benefits over conventional therapy in preventing cardiac remodeling [19]. After six months, the RES group exhibited smaller left atrial size, lower E/e ratio, higher left ventricular global longitudinal strain, and reduced biomarkers of cardiac fibrosis (procollagen type I C-peptide and galectin-3) compared to the control group (no RES supplementation with standardized antihypertensive therapy alone) [19]. However, no significant differences in left ventricular structure, arterial stiffness, or BP were observed between the groups [19]. Given that left ventricular hypertrophy and myocardial fibrosis induced by hypertension are key risk factors for heart failure, further research is warranted to explore the potential benefits of RES in improving outcomes in patients with hypertension.

In addition to micronutrients, dietary patterns are important elements of a healthy diet. A longitudinal study in China investigated the relationship between dietary patterns and the incidence of hypertension among Chinese adults between 1991 and 2018. Using data from the China Health and Nutrition Survey, researchers identified various dietary patterns through factor analysis and assessed their impact on hypertension [20]. Key findings indicated that modern patterns, rich in fruits, dairy products, cakes, cookies, and pastries, were associated with a decrease in SBP (adjusted β = −0.51; 95% CI −0.86 and −0.16; *P* < 0.01) [20]. Conversely, the meat pattern: unhealthy dietary patterns with high loadings of poultry, organ meats and other livestock meat, were positively associated with increased in diastolic blood pressure (DBP) (adjusted β = 0.31; 95% CI 0.08–0.53; *P* < 0.01) and risk of hypertension (adjusted OR 1.14, 95% CI 1.03–1.24) [20]. Furthermore, the southern pattern, rich in rice, vegetables, and pork, which is the predominant dietary pattern in China, did not significantly affect BP or the risk of hypertension [20]. Over the last several decades, dietary pattern has been dramatically changing and meat consumption has been increasing in East Asia, not only in China, but also in Japan [21, 22]. Not only that, despite continuous efforts in the prevention and management of hypertension, the global number of hypertensive patients has doubled between 1990 and 2019 [23]. Since the relationship between nutrition and hypertension is influenced by changes in dietary habits and disease structures, it is necessary to evaluate and develop healthy dietary patterns that practically fit the times and the region.

Salt reduction is an important nonpharmacological treatment for hypertension, and initiatives to reduce salt intake have been implemented worldwide. However, salt consumption in the world remains high, and no study evaluated the public interest in salt reduction by analyzing Internet research activity. Suzuki et al. explored how the Japanese public’s attention to salt reduction has changed over time by analyzing Google Trends [24]. This study retrospectively analyzed data from Google Trends from 2004 to 2021. The research found that the relative search volumes (RSVs) for “salt reduction” significantly increased in 2021 compared to 2004 (13.8 ± 9.3% to 70.8 ± 10.9%, *r* = 0.92, *P* < 0.001) [24]. Analyzing large amounts of real-time Internet research activity could provide valuable insights for designing more effective public health interventions aimed at increasing awareness and promoting salt reduction. In addition to this study, numerous other significant studies on salt intake and BP were published in high-impact international journals in 2023. The findings are introduced in the following sections.

## Salt intake and blood pressure research findings reported in 2023

Several unique interventional studies that examined the impact of salt reduction on BP change have been reported in leading journals. Although reducing salt intake is generally recommended to lower BP, variability in individual responses makes personalized treatment challenging. The concept of salt sensitivity of BP (SSBP) refers to a physiological condition in which an individual’s BP fluctuates significantly in response to changes in dietary sodium intake [25]. Individuals with SSBP experienced a marked decrease in BP when sodium intake was reduced and an increase in BP when sodium intake was increased. This phenomenon highlights the variability in the effects of sodium on BP among different individuals. It has been reported that around 50% of individuals with hypertension and 25% of those without hypertension exhibit SSBP [25, 26]. Nonetheless, most studies have examined the effects of salt reduction on BP management, excluding those on antihypertensive medications; thus, the benefits of sodium reduction in such populations uncertain. Gupta et al. performed a crossover trial that investigated the impact of dietary sodium intake on BP among middle-aged to elderly adults, including those with normotension, controlled hypertension, uncontrolled hypertension, and untreated hypertension [27]. This crossover study involved 213 participants aged 50 to 75 years who followed high-sodium (adding 2200 mg/day sodium to the usual diet) and low-sodium (total 500 mg /day) diets for one week each. The results indicated that reducing dietary sodium significantly lowered BP, regardless of hypertension status or antihypertensive medication use [27]. Moreover, this finding was not altered across all subgroups (age, sex, race, baseline BP, diabetes, and body mass index) [27]. The median change in mean arterial pressure within individuals between high- and low-sodium diets was 4 mm Hg (IQR, 0–8 mm Hg; *P* < 0.001), and 73.4% of participants experiencing a reduction in mean arterial pressure on a low-sodium diet. Furthermore, this study did not report excess adverse events [27]. The results of this study suggested that the reduction in BP achieved through dietary sodium reduction was similar to that of a commonly prescribed first-line antihypertensive drug, regardless of antihypertensive medication use across subgroups.

Another study investigated the impact of a community-based intervention to reduce salt intake by educating home cooks in China [28]. Conducted as a cluster-RCT, the study included 60 communities across six provinces, with interventions lasting 12 months [28]. A total of 30 communities with 786 participants were allocated to the intervention group, and 30 communities with 790 participants were allocated to the control group. Participants in the intervention group received supportive environments for salt reduction, educational sessions, and salt intake monitoring. The control group did not receive any interventions. The main outcome was the change in salt intake measured by 24-hour urinary sodium excretion [28]. Results showed a significant reduction in urinary sodium excretion in the intervention group by 336.8 mg/day (95% CI 127.9–545.7), equivalent to salt intake of 0.9 g/day, compared to the control groups, along with reductions in systolic and diastolic BP by 1.98 mm Hg (95% CI −3.54 and −0.41) and 1.05 mm Hg (95% CI −2.01 and −0.10), respectively. The intervention also improved participants’ knowledge, attitudes, and behaviors regarding salt reduction [28]. This study presents novel scientific evidence regarding the role of home cooks in salt reduction interventions, with the potential for broad application in China and other countries, where home cooking constitutes a significant source of salt intake.

Salt substitution has attracted substantial attention as a key strategy for salt reduction. The latest meta-analysis results on the cardiovascular disease prevention effects of salt substitution were reported [29]. This meta-analysis examined the long-term (≥ 6 months) effects of salt substitution (potassium-enriched, sodium-reduced salt substitutes: regardless of any sodium to potassium ratio) on cardiovascular outcomes by synthesizing data from 16 RCTs. Findings revealed that salt substitute may reduce risk for all-cause mortality with rate ratio (RR) of 0.88 (95% CI 0.82–0.93) and cardiovascular mortality with RR of 0.83 (95% CI 0.73–0.95). These risk reductions correspond to 5 (95% CI 3–7) fewer all-cause deaths per 1000 participants per year and 3 (95% CI 1–5) fever CVD deaths per 1000 participants per year with a higher-than-average baseline CVD risk. Nonetheless, all included RCTs to evaluate the preventive effects of salt substitutes on all-cause mortality and CVD mortality were performed in China or Taiwan, and all included populations of older age (mean 64 years) and/or with a higher-than-average risk for CVD [29]. Therefore, the indirectness of the results was very serious, and this fact lowered the certainty of evidence to low, as assessed by the Grading of Recommendations Assessment, Development and Evaluation (GRADE) approach (high, moderate, low, or very low certainty) [30]. They also revealed that salt substitution significantly reduces SBP and DBP by 5.12 mmHg and 1.56 mmHg, respectively [29]. However, the certainty of evidence was very low and the results showed substantial heterogeneity (I^2^; 60–81%), which can be partly explained by the age of the participants, follow-up duration, and percentage of potassium chloride in the salt substitute [29]. Regarding the risk of adverse events, evidence remains highly uncertain about the impact of salt substitution on serious adverse outcomes or harm (RR 1.04, 95% CI 0.87–1.25, I² = 21%; evidence of very low certainty) [29]. Although the grade of the evidence supporting the mortality-reducing effects of salt substitution is not high, particularly among Western populations, this study suggests that salt substitution represents a promising and scalable non-pharmacological intervention that could reduce mortality, especially among populations such as those adhering to an East Asian diet or those at a high risk for CVD.

Regarding the cost-effectiveness of salt substitutes, the Salt Substitute and Stroke Study (SSaSS) provided evidence that salt substitutes are both effective and cost-saving in reducing the risk of major CVD events and all-cause mortality among individuals at high CVD risk [31, 32]. However, no evidence exists regarding the health benefits and cost-effectiveness of salt substitution, specifically for older adults in elderly care residential facilities. A cluster randomized study evaluated the cost-effectiveness of using salt substitutes (consisted of 62.5% sodium chloride, 25% potassium chloride, and 12.5% dried food ingredients) and implementing salt-supply restrictions in elderly care facilities in China. This study involved 48 elderly care facilities divided into four groups: salt substitute only, salt supply restriction only, both interventions combined, and a control group with no interventions [33]. The incremental cost-utility ratio was evaluated as the additional average cost incurred per quality-adjusted life-year (QALY). 1612 participants (males 76.3%, mean age 71.0 ± 9.5 years) were enrolled in this study. Replacing regular salt with a salt substitute led to a reduction in mean SBP by 7.14 mmHg (95% CI 3.79–10.48), a decrease in hypertension prevalence by 5.09% (95% CI 0.37–9.80), and a reduction in major adverse cardiovascular events (MACEs) by 2.27% (95% CI 0.09–4.45) [33]. After the 2-year intervention, the mean cost for the salt substitute group was $25.95 lower than that for the regular salt group, primarily due to significant savings in health care costs associated with MACEs (mean [SD], $72.88 [$9.11] vs. $111.18 [$13.90]) [33]. On the other hand, the salt restriction strategy did not reveal significant reduction. This study also reported that, if the salt substitution strategy was implemented across all elderly care facilities in China, estimated 48,101 MACEs and 107,857 cases of hypertension could be averted, resulting in an estimated savings of $54,982,278 within the first two years [33]. In an aging society, elderly care facilities are a crucial component of the social infrastructure. The demonstrated benefits of using salt substitutes in these facilities, in terms of improved CVD outcomes and economic efficiency, could be highly valuable for informing national public health strategies.

The Dietary Approaches to Stop Hypertension (DAHS) diet, which is rich in fruits, vegetables, and low-fat dairy and low in red / processed meats and sugar-sweetened beverages, reduces BP [34], and it is recommended to prevent CVD [35]. In the DASH-Sodium trial, the DASH diet lowered BP at all sodium levels (high, moderate, and low) compared to the control diet. The reduction in BP was most significant when the DASH diet was paired with low-sodium intake, as opposed to following the DASH diet or low-sodium intake alone [36]. However, the mechanisms underlying these effects have not been fully elucidated. Kim et al. explored the metabolomic profiles linked to BP reduction in participants of two randomized feeding studies: the Dietary Approaches to Stop Hypertension (DASH) and DASH-Sodium trials [37]. Utilizing metabolomic profiling of serum and urine samples, they identified specific metabolites associated with BP following dietary interventions by evaluating the interactions between dietary interventions and metabolites [37]. The study found 65 significant interactions between metabolites and BP changes, and 42 unique metabolites (nine serum metabolites and 33 urine metabolites) were associated with BP [33]. In the DASH trial, serum tryptophan betaine was associated with decrease in DBP among participants allocated to the DASH diet but not among those on the control diet. In the DASH-sodium trial, urinary levels of N-methylglutamate and proline derivatives (including stachydrine, 3-hydroxystachydrine, N-methylproline, and N-methylhydroxyproline) were associated with decrease in either SBP or DBP in participants consuming the DASH diet, with no such association observed in the control group [37]. These findings suggest that these metabolites highlight the biochemical pathways through which the DASH diet exerts its BP-lowering effects, and the results of the study provides deeper insights on planning dietary strategies to lower BP.

**Fig. 1:**
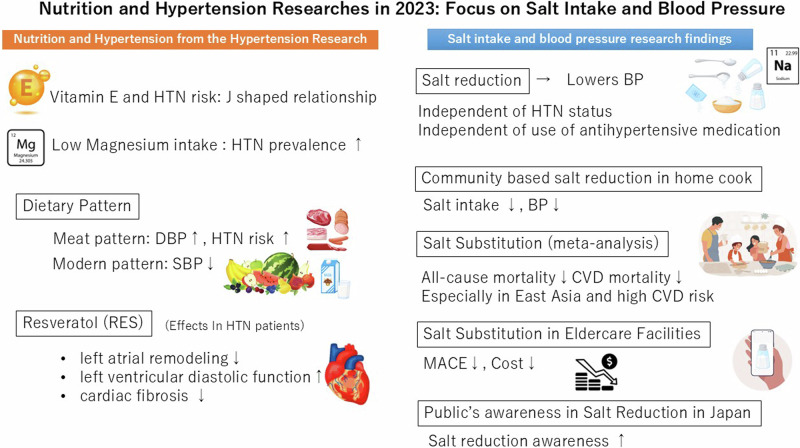
Nutrition and Hypertension Researches in 2023: Focus on Salt Intake and Blood Pressure. HTN hypertension, DBP diastolic blood pressure, SBP systolic blood pressure, CVD cardiovascular disease, MACE Major adverse cardiovascular event

The new findings from nutritional studies in 2023 provide insight into dietary modifications, particularly sodium reduction, for managing and preventing hypertension (Fig. 1). Nutritional interventions play a crucial role in hypertension management as non-pharmacological treatments. Further research is needed to elucidate effective nutritional approaches tailored to individual and population characteristics.

## References

[1] Han M, Zhang Y, Fang J, Sun M, Liu Q, Ma Z, et al. Associations between dietary magnesium intake and hypertension, diabetes, and hyperlipidemia. Hypertens Res. 2024;47:331–41. 37821564 10.1038/s41440-023-01439-z

[2] Zhang X, Li Y, Del Gobbo LC, Rosanoff A, Wang J, Zhang W, et al. Effects of magnesium supplementation on blood pressure: a meta-analysis of randomized double-blind placebo-controlled trials. Hypertension. 2016;68:324–33. 27402922 10.1161/HYPERTENSIONAHA.116.07664

[3] Yoshimura M, Oshima T, Matsuura H, Ishida T, Kambe M, Kajiyama G. Extracellular Mg2+ inhibits capacitative Ca2+ entry in vascular smooth muscle cells. Circulation. 1997;95:2567–72. 9184588 10.1161/01.cir.95.11.2567

[4] Fujita T, Ito Y, Ando K, Noda H, Ogata E. Attenuated vasodilator responses to Mg2+ in young patients with borderline hypertension. Circulation. 1990;82:384–93. 2372889 10.1161/01.cir.82.2.384

[5] Blache D, Devaux S, Joubert O, Loreau N, Schneider M, Durand P, et al. Long-term moderate magnesium-deficient diet shows relationships between blood pressure, inflammation and oxidant stress defense in aging rats. Free Radic Biol Med. 2006;41:277–84. 16814108 10.1016/j.freeradbiomed.2006.04.008

[6] Perales AJ, Torregrosa G, Salom JB, Miranda FJ, Alabadí JA, Monleón J, et al. In vivo and in vitro effects of magnesium sulfate in the cerebrovascular bed of the goat. Am J Obstet Gynecol. 1991;165:1534–8. 1957890 10.1016/0002-9378(91)90401-c

[7] Zhang Y, Yang S, Wu Q, Ye Z, Zhou C, Liu M, et al. Dietary vitamin E intake and new-onset hypertension. Hypertens Res. 2023;46:1267–75. 36609495 10.1038/s41440-022-01163-0

[8] Ashor AW, Siervo M, Lara J, Oggioni C, Afshar S, Mathers JC. Effect of vitamin C and vitamin E supplementation on endothelial function: a systematic review and meta-analysis of randomised controlled trials. Br J Nutr. 2015;113:1182–94. 25919436 10.1017/S0007114515000227

[9] Chiu HF, Venkatakrishnan K, Golovinskaia O, Wang CK. Impact of micronutrients on hypertension: evidence from clinical trials with a special focus on meta-analysis. Nutrients. 2021;13:588. 33578935 10.3390/nu13020588 PMC7916651

[10] Emami MR, Safabakhsh M, Alizadeh S, Asbaghi O, Khosroshahi MZ. Effect of vitamin E supplementation on blood pressure: a systematic review and meta-analysis. J Hum Hypertens. 2019;33:499–507. 30846828 10.1038/s41371-019-0192-0

[11] Traber MG, Stevens JF. Vitamins C and E: beneficial effects from a mechanistic perspective. Free Radic Biol Med. 2011;51:1000–13. 21664268 10.1016/j.freeradbiomed.2011.05.017 PMC3156342

[12] Wu D, Liu L, Meydani M, Meydani SN. Vitamin E increases production of vasodilator prostanoids in human aortic endothelial cells through opposing effects on cyclooxygenase-2 and phospholipase A2. J Nutr. 2005;135:1847–53. 16046707 10.1093/jn/135.8.1847

[13] Pearson P, Lewis SA, Britton J, Young IS, Fogarty A. The pro-oxidant activity of high-dose vitamin E supplements in vivo. BioDrugs. 2006;20:271–3. 17025373 10.2165/00063030-200620050-00002

[14] Keaney JF Jr., Gaziano JM, Xu A, Frei B, Curran-Celentano J, Shwaery GT, et al. Low-dose alpha-tocopherol improves and high-dose alpha-tocopherol worsens endothelial vasodilator function in cholesterol-fed rabbits. J Clin Invest. 1994;93:844–51. 8113416 10.1172/JCI117039 PMC293946

[15] Brown BG, Zhao XQ, Chait A, Fisher LD, Cheung MC, Morse JS, et al. Simvastatin and niacin, antioxidant vitamins, or the combination for the prevention of coronary disease. N Engl J Med. 2001;345:1583–92. 11757504 10.1056/NEJMoa011090

[16] Thandapilly SJ, Wojciechowski P, Behbahani J, Louis XL, Yu L, Juric D, et al. Resveratrol prevents the development of pathological cardiac hypertrophy and contractile dysfunction in the SHR without lowering blood pressure. Am J Hypertens. 2010;23:192–6. 19942861 10.1038/ajh.2009.228

[17] Dolinsky VW, Chakrabarti S, Pereira TJ, Oka T, Levasseur J, Beker D, et al. Resveratrol prevents hypertension and cardiac hypertrophy in hypertensive rats and mice. Biochim Biophys Acta. 2013;1832:1723–33. 23707558 10.1016/j.bbadis.2013.05.018

[18] Gupta PK, DiPette DJ, Supowit SC. Protective effect of resveratrol against pressure overload-induced heart failure. Food Sci Nutr. 2014;2:218–29. 24936291 10.1002/fsn3.92 PMC4048607

[19] Zheng X, Hai J, Yang Y, Zhang C, Ma X, Kong B, et al. Effects of resveratrol supplementation on cardiac remodeling in hypertensive patients: a randomized controlled clinical trial. Hypertens Res. 2023;46:1493–503. 36854725 10.1038/s41440-023-01231-z

[20] Zhang J, Du W, Huang F, Li L, Bai J, Wei Y, et al. Longitudinal study of dietary patterns and hypertension in adults: China Health and Nutrition Survey 1991–2018. Hypertens Res. 2023;46:2264–71. 37337099 10.1038/s41440-023-01322-x PMC10550817

[21] OECD FaAOotUN. OECD‑FAO Agricultural Outlook 2021‑2030. 2021.

[22] Ministry of Agriculture FaF. Food Balance Sheet. https://www.maff.go.jp/j/zyukyu/fbs/.

[23] NCD Risk Factor Collaboration (NCD-RisC). Worldwide trends in hypertension prevalence and progress in treatment and control from 1990 to 2019: a pooled analysis of 1201 population-representative studies with 104 million participants. Lancet2021;398:957–80. 34450083 10.1016/S0140-6736(21)01330-1 PMC8446938

[24] Suzuki T, Kishi T, Ishida M, Rewley J, Node K, Mizuno A. The time trend of information seeking behavior about salt reduction using Google Trends: infodemiological study in Japan. Hypertens Res. 2023;46:1886–91. 37106044 10.1038/s41440-023-01283-1

[25] Elijovich F, Weinberger MH, Anderson CA, Appel LJ, Bursztyn M, Cook NR, et al. Salt sensitivity of blood pressure: a scientific statement from the American Heart Association. Hypertension. 2016;68:e7–e46. 27443572 10.1161/HYP.0000000000000047

[26] Kurtz TW, DiCarlo SE, Pravenec M, Morris RC Jr. An appraisal of methods recently recommended for testing salt sensitivity of blood pressure. J Am Heart Assoc. 2017;6:e005653. 28365569 10.1161/JAHA.117.005653 PMC5533040

[27] Gupta DK, Lewis CE, Varady KA, Su YR, Madhur MS, Lackland DT, et al. Effect of dietary sodium on blood pressure: a crossover trial. JAMA. 2023;330:2258–66. 37950918 10.1001/jama.2023.23651 PMC10640704

[28] Zhang X, Zhang P, Shen D, Li Y, He FJ, Ma J, et al. Effect of home cook interventions for salt reduction in China: cluster randomised controlled trial. BMJ. 2023;382:e074258. 37620015 10.1136/bmj-2022-074258 PMC10448250

[29] Greenwood H, Barnes K, Clark J, Ball L, Albarqouni L. Long-term effect of salt substitution for cardiovascular outcomes : a systematic review and meta-analysis. Ann Intern Med. 2024;177:643–55. 38588546 10.7326/M23-2626

[30] Guyatt GH, Oxman AD, Vist GE, Kunz R, Falck-Ytter Y, Alonso-Coello P, et al. GRADE: an emerging consensus on rating quality of evidence and strength of recommendations. BMJ. 2008;336:924–6. 18436948 10.1136/bmj.39489.470347.AD PMC2335261

[31] Neal B, Wu Y, Feng X, Zhang R, Zhang Y, Shi J, et al. Effect of salt substitution on cardiovascular events and death. N Engl J Med. 2021;385:1067–77. 34459569 10.1056/NEJMoa2105675

[32] Li KC, Huang L, Tian M, Di Tanna GL, Yu J, Zhang X, et al. Cost-effectiveness of a household salt substitution intervention: findings From 20 995 Participants of the salt substitute and stroke study. Circulation. 2022;145:1534–41. 35311346 10.1161/CIRCULATIONAHA.122.059573

[33] Lai X, Yuan Y, Wang H, Zhang R, Qiao Q, Feng X, et al. Cost-effectiveness of salt substitute and salt supply restriction in eldercare facilities: the DECIDE-salt cluster randomized clinical trial. JAMA Netw Open. 2024;7:e2355564. 38345818 10.1001/jamanetworkopen.2023.55564 PMC10862151

[34] Appel LJ, Moore TJ, Obarzanek E, Vollmer WM, Svetkey LP, Sacks FM, et al. A clinical trial of the effects of dietary patterns on blood pressure DASH Collaborative Res Group. N Engl J Med. 1997;336:1117–24. 9099655 10.1056/NEJM199704173361601

[35] Lichtenstein AH, Appel LJ, Vadiveloo M, Hu FB, Kris-Etherton PM, Rebholz CM, et al. 2021 dietary guidance to improve cardiovascular health: a scientific statement from the American Heart Association. Circulation. 2021;144:e472–e87. 34724806 10.1161/CIR.0000000000001031

[36] Sacks FM, Svetkey LP, Vollmer WM, Appel LJ, Bray GA, Harsha D, et al. Effects on blood pressure of reduced dietary sodium and the Dietary Approaches to Stop Hypertension (DASH) diet DASH-Sodium Collaborative Res Group. N. Engl J Med. 2001;344:3–10. 11136953 10.1056/NEJM200101043440101

[37] Kim H, Appel LJ, Lichtenstein AH, Wong KE, Chatterjee N, Rhee EP, et al. Metabolomic profiles associated with blood pressure reduction in response to the DASH and DASH-sodium dietary interventions. Hypertension. 2023;80:1494–506. 37161796 10.1161/HYPERTENSIONAHA.123.20901 PMC10262995

